# Influenza Viral Vectors Expressing Two Kinds of HA Proteins as Bivalent Vaccine Against Highly Pathogenic Avian Influenza Viruses of Clade 2.3.4.4 H5 and H7N9

**DOI:** 10.3389/fmicb.2018.00604

**Published:** 2018-04-04

**Authors:** Jinping Li, Guangyu Hou, Yan Wang, Suchun Wang, Cheng Peng, Xiaohui Yu, Wenming Jiang

**Affiliations:** ^1^China Animal Health and Epidemiology Center, Qingdao, China; ^2^Shanghai Entry-Exit Inspection and Quarantine Bureau, Shanghai, China

**Keywords:** influenza virus vector, non-structural protein, bivalent vaccine, H5, H7N9, avian influenza virus

## Abstract

The H5 and H7N9 subtypes of highly pathogenic avian influenza viruses (HPAIVs) in China pose a serious challenge to public health and the poultry industry. In this study, a replication competent recombinant influenza A virus of the Í5N1 subtype expressing the H7 HA1 protein from a tri-cistronic NS segment was constructed. A heterologous dimerization domain was used to combine with the truncated NS1 protein of 73 amino acids to increase protein stability. H7 HA1, nuclear export protein coding region, and the truncated NS1 were fused in-frame into a single open reading frame via 2A self-cleaving peptides. The resulting PR8-H5-NS1(73)H7 stably expressed the H5 HA and H7 HA1 proteins, and exhibited similar growth kinetics as the parental PR8-H5 virus *in vitro*. PR8-H5-NS1(73)H7 induced specific hemagglutination inhibition (HI) antibody against H5, which was comparable to that of the combination vaccine of PR8-H5 and PR8-H7. The HI antibody titers against H7 virus were significantly lower than that by the combination vaccine. PR8-H5-NS1(73)H7 completely protected chickens from challenge with both H5 and H7 HPAIVs. These results suggest that PR8-H5-NS1(73)H7 is highly immunogenic and efficacious against both H5 and H7N9 HPAIVs in chickens.

**Highlights**:

- PR8-H5-NS1(73)H7 simultaneously expressed two HA proteins of different avian influenza virus subtypes.

- PR8-H5-NS1(73)H7 was highly immunogenic in chickens.

- PR8-H5-NS1(73)H7 provided complete protection against challenge with both H5 and H7N9 HPAIVs.

## Introduction

H5N1 avian influenza viruses (AIVs) have circulated in more than 60 countries and have caused huge economic losses to the poultry industry worldwide (Swayne, 2012). Of note, the H5N1 AIVs have become enzootic in poultry and wild birds in China (including Hong Kong special administrative region [SAR]), Bangladesh, eastern India, Indonesia, Vietnam, and Egypt (Swayne et al., 2011). Sequence analyses of the HA genes have shown that H5 viruses have evolved into diverse clades and subclades (Li et al., 2010). Viruses in clades 2.3.2.1 and 2.3.4.4 continue to cocirculate in wild birds and poultry in several countries, while clade 7.2 viruses have been detected in chickens in several provinces in Northern China (Li et al., 2010). Recent molecular epidemiological surveys of AIVs have detected no clade 7.2 viruses and significant decreases in circulating clade 2.3.2.1 viruses since 2015 (data not published). Currently, clade 2.3.4.4 viruses are the dominant epidemic strains.

Vaccination is an important strategy to control H5N1 AIV infection of poultry in mainland China, Hong Kong SAR, Vietnam, Indonesia, and Egypt (Swayne, 2012; Swayne et al., 2014). The vaccine strains used in China have been updated several times since 2004 to ensure an antigenic match between the vaccines and the prevalent strains (Li et al., 2014). The inactivated H5N1 vaccine produced from the vaccine strain Re8, which was generated by reverse genetics and contains the HA and NA genes of a clade 2.3.4.4 virus, A/chicken/Guizhou/4/2013(H5N1), has been widely used to control clade 2.3.4.4 AIVs in mainland China and Hong Kong since 2016 (Zeng et al., 2016).

Since 2013, novel H7N9 viruses have been isolated from live poultry markets in China and have always shown low pathogenicity in poultry. However, since December 2016, H7N9 viruses with novel polybasic amino acid sequences at the HA cleavage sites, corresponding with increased pathogenicity in poultry, have been detected in both human patients and chickens, and were found to be highly pathogenic in chickens (Wang et al., 2017). Since March 2017, highly pathogenic H7N9 viruses have caused 26 outbreaks in ten provinces in China, which have resulted in huge economic losses to the poultry industry. To address this problem, the government of China implemented a mass vaccination program of poultry with an inactivated H7N9 vaccine produced from the vaccine strain H7 Re1, which was generated by reverse genetics and contains the HA and NA genes of A/pigeon/Shanghai/S1069/2013(H7N9). The combination vaccine of H5 Re-8 and H7 Re1 has been used to control highly pathogenic H5 and H7N9 AIVs throughout China since August 2017. Despite the availability of this efficacious combination vaccine, vaccine production requires more ECEs, an alternative vaccine (i.e., a bivalent recombinant vaccine expressing both corresponding antigens) would be more advantageous.

The influenza A virus is a segmented RNA virus, with a genome consisting of eight gene fragments. Of these, the smallest fragment (NS), encoding for the non-structural protein 1 (NS1) and nuclear export protein (NEP), is a efficient target for genetic manipulation since NS1 could tolerate insertion of foreign genes exceeding its own length (Kittel et al., 2004). Thus, in this study, the open reading frame of NS1 was used for insertion of the mature HA1 sequence of the H7N9 virus with the A/Puerto Rico/8/34 (H1N1) strain as the backbone to obtain influenza A virus vectors expressing the mature HA1 sequence of H7N9 in a fusion protein with 73 N-terminal amino acid residues of NS1 and expressing the HA and NA proteins of H5, which replaced the corresponding proteins of the PR8 virus. The aim of the present study was to evaluate the immunogenicity and protective efficacy of the bivalent vaccine in chickens against challenge with both clade 2.3.4.4 H5 and H7N9 highly pathogenic AIVs (HPAIVs).

## Materials and Methods

### Ethics Statements and Facility

The study protocol was approved by the Ethics Committee of China Animal Health and Epidemiology Center (Qingdao, China) and conducted in strict accordance with the recommendations outlined in the Guide for the Care and Use of Laboratory Animals of the Ministry of Science and Technology of the People’s Republic of China. The approval of recombinant work with HPAI viruses was also granted. All experiments with lethal H5 and H7 viruses were performed in a biosafety level 3 facility, and all animal experiments were performed in high-efficiency particulate air-filtered isolators at the China Animal Health and Epidemiology Center.

### Viruses and Cells

The HPAIVs A/chicken/Fujian/5/2016(H5N6) (FJ/5) (clade 2.3.4.4) and A/chicken/Guangdong/RZ/2017(H7N9) (GD/RZ) were isolated from dead chickens, and propagated in 10-day-old specific-pathogen-free (SPF) embryonated chicken eggs (ECEs). The intravenous pathogenicity index of these HPAIVs was 3.00 as determined in ten 6-week-old SPF chickens that were intravenously inoculated with 0.1 mL of a 1/10 dilution of the fresh infectious allantoic fluid of FJ/5 and GD/RZ, according to the OIE (World Organization for Animal Health) manual (OIE, 2008). Human embryonic kidney (HEK) 293T cells were maintained in Dulbecco’s modified Eagle medium supplemented with 10% fetal calf serum and maintained at 37°C under an atmosphere of 5% CO_2_.

### Construction of the Plasmid pHW-NS1(73)-H7 HA1-NEP

The coding sequence of Dmd/FMDV-2A (Foot-and-mouth disease virus, FMDV) was synthesized (Sangon Biotech Co. Ltd., Shanghai, China) and cloned into the pcDNA3 vector by the NotI and XbaI restriction sites. The sequence coding for the first 73 amino acids of NS1 was amplified by polymerase chain reaction (PCR) from the pHW198-NS plasmid (Hoffmann et al., 2002) and cloned 5′ of and in frame with the Dmd/FMDV-2A by the BamHI and EspEI restriction sites. This NS(73)-Dmd/FMDV-2A was then cloned into the pHW2000 plasmid by the BamHI and MunI restriction sites.

The mature HA1 coding sequence (derived from GD/RZ) was cloned 3′ of and in frame with the FMDV-2A self-cleaving sequence by the BglII and EcoRI restriction sites. The porcine teschovirus-1 (PTV-1) 2A self-cleaving sequence was fused to the NEP coding sequence (by fusion PCR) and cloned 3′ of and in frame with the mature HA1 coding sequence by the EcoRI and BstEII restriction sites.

### Generation of Recombinant Viruses

All viruses were rescued with a standard reverse genetics method using eight bidirectional plasmids (pHW2000) (Hoffmann et al., 2000). Briefly, HEK 293T cells were co-transfected with 0.8 μg of each of the five pHW-plasmids (pHW191-PB2, pHW192-PB1, pHW193-PA, pHW195-NP, and pHW197-M) and pHW-NS1(73)-H7 HA1-NEP, as well as the HA and NA genes of the FJ/5 virus using Lipofectamine 3000 transfection reagent (Life Technologies, Carlsbad, CA, United States). The HA protein sequence of the FJ/5 virus was modified by removal of the multi-basic amino acid motif from RERRRKRG to RETRG located in the HA cleavage site. After 24 h, TPCK (L-1-tosylamide-2-phenylethyl chloromethyl ketone)-treated trypsin (Sigma-Aldrich Corporation, St. Louis, MO, United States) was added at a final concentration of 2 μg/mL. After 72 h, the supernatants of transfected cells were collected and inoculated into 10-day-old SPF ECEs which were incubated at 37°C for 72 h.

Similarly, recombinant PR8-H5 and PR8-H7 viruses were also rescued using pHW198-NS with the HA protein sequence of the FJ/5 or GD/RZ virus that was modified by removal of the multi-basic amino acid motif located in the HA cleavage site. Vaccine batches were produced in SPF ECEs after five egg passages of viral constructs.

### Determination of the 50% Embryo Infectious Dose (EID_50_) of the Viruses

The infectious titers of the viruses were determined using 10-day old SPF ECEs by standard methods. Viral suspensions (10^-1^ – 10^-9^ dilutions) were prepared in phosphate-buffered saline (PBS) (pH 7.2) and the allantoic cavities of five ECEs were infected with 0.1 mL of each dilution. The ECEs were incubated at 37°C at a relative humidity of 60% for 72 h. The viral titers were determined by the hemagglutination assay as described in the WHO Manual on Animal Influenza Diagnosis and Surveillance. The assay was carried out in triple replicates. Viral titers were calculated and expressed as mean EID_50_/ml (Log10) ± standard errors.

### Growth Kinetics *in Vitro*

Ten-day-old SPF ECEs were infected with 10^4^ EID_50_ of the PR8-H5, PR8-H7, or PR8-H5-NS1(73)H7 virus. Samples of allantoic fluid from five eggs infected with each virus were collected at 0, 4, 8, 12, 24, 36, and 48 h. The viral titers of the samples were determined by EID_50_ analysis.

### Genetic Stability of the PR8-H5-NS1(73)H7 Virus

Ten consecutive passages of the PR8-H5-NS1(73)H7 virus were propagated in 10-day-old ECEs for genetic stability testing. The allantoic cavities of the ECEs were inoculated with 10^-4^ dilutions of the virus. The genetic stability of the viral constructs was confirmed by reverse transcription (RT)-PCR with primers specific to the NS segment (sense 5′-GTA GAT TGC TTT CTT TGG-3′ and antisense 5′-CTA AAT AAG CTG AAA CGA-3′). At passages 1, 3, 5, and 10, the size of the NS amplicon was compared to that of the pHW plasmid encoding the corresponding gene. The NS1-fusion protein encoding genes of the viral constructs were sequenced at passages 1, 3, 5, and 10 using the Sanger method with the commercial Prism BigDye^TM^ Terminator v3.1 kit (Applied Biosystems, Foster City, CA, United States) on an automatic sequencer (Genetic Analyser 3730XL; Applied Biosystems).

### Determination of H7 HA1 Protein Expression With Western Blot Analysis

ECEs was inoculated with 10^4^ EID_50_ of the PR8-H5, PR8-H7, or PR8-H5-NS1(73)H7 virus. After 72 h of incubation at 37°C, allantoic fluid samples from uninfected ECEs and infected ECEs were purified by ultracentrifugation through a 20% sucrose cushion, mixed with Laemmli buffer containing β-mercaptoethanol, and boiled for 5 min. The proteins were separated with sodium dodecyl sulfate polyacrylamide gel electrophoresis and the targeted proteins were visualized with western blotting using an anti-H5 or anti-H7 antibody. The antisera were prepared by immunization of BALB/c mice with the HA proteins of the FJ/5 or GD/RZ virus. For western blot analysis, the proteins were transferred to nitrocellulose membranes, which were blocked in blocking buffer (150 mÌ NaCl, 20 mM Tris–HCl, pH 7.5, containing 5% skimmed milk powder) at room temperature for 1 h. The blot was probed with mouse anti-H5N1 or anti-H7N9 serum at a dilution 1:2000 in blocking buffer containing 0.1% Tween-20 at room temperature for 2 h. Following three washes with Tris-buffered saline with Tween-20 (TBST) buffer (150 mM NaCl, 20 mM Tris–HCl, pH 7.5, 0.1% Tween-20), the blot was incubated with horseradish peroxidase-conjugated goat-anti-mouse IgG antibody (Boster, Wuhan, China) for 1–2 h at room temperature. Then, the membranes were washed three times for 10 min each with TBST buffer and the bands were visualized with the addition of 5-bromo-4-chloro-3-indolyl-phosphate/nitro blue tetrazolium color development substrate (Sigma-Aldrich Corporation).

### Vaccine Preparation

Vaccine samples were prepared from viral constructs of the PR8-H5, PR8-H7, and PR8-H5-NS1(73)H7 viruses that were accumulated in 10-day-old ECEs at 37°C for 72 h. Formalin (final concentration, 0.1%) was added to inactivate the allantoic suspensions of viral constructs, which were then incubated at 4°C for 72 h. Inactivated viruses were concentrated, purified through a 10–50% sucrose density gradient, and resuspended in PBS. The obtained allantoic suspensions of the PR8-H5 and PR8-H7 viruses were pooled at a 1:1 ratio to obtain the combination vaccine formulation. Then, the oil-adjuvant whole-virus inactivated vaccine was prepared with viral constructs (the inactivated virus mixed with mineral oil adjuvant at 1:2 (Vol/Vol) and emulsified) and the HA protein content in the final vaccine preparation is about 9.24 mg/ml, which was quantified as previous described (Tian et al., 2005).

### Vaccination and Challenge Test

Groups of 20 3-week-old white Leghorn SPF chickens were injected intramuscularly (i.m.) with 0.3 mL of formalin-inactivated vaccine of the bivalent PR8-H5-NS1(73)H7 or the combination of PR8-H5 and PR8-H7. Groups of 20 chickens were injected i.m. with 0.3 mL of univalent vaccine of PR8-H5 or PR8-H7. Groups of 30 chickens were injected i.m. with 0.3 mL of PBS as a control. Three weeks after vaccination, serum samples were collected from each chicken for antibody detection. Then, the birds were challenged intranasally with 10^6^ EID_50_ of the lethal H5 or H7 virus. Oropharyngeal and cloacal swabs were collected on days 3 and 5 post-challenge (p.c.) for virus isolation and titration in eggs. All birds were observed for signs of disease or death for 10 days p.c.

### Antibody Detection Using the HI Assay

Specific antibodies in the chicken sera were detected using the HI assay as described previously (Jiang et al., 2014). Briefly, the sera were inactivated by incubation at 56°C for 30 min. Then, the sera were twofold serially diluted with PBS, and incubated with four hemagglutination units of the target influenza virus for 30 min. This was followed by adding equal volumes of fresh 1.0% (v/v) chicken red blood cells and further incubation of 30 min. The sample HI titer was defined as the reciprocal of the highest dilution that completely inhibits the agglutination.

### Statistical Analysis

Statistical analyses were performed by using SPSS 20.0 for Windows. Two-way ANOVA was performed on data. Data in graphs or tables are presented as means with their standard errors of the means. *p* > 0.05 was considered as non-significant, *p* < 0.05 were considered as significant.

## Results

### Generation of Recombinant Influenza A Virus Expressing the H7 HA1 Protein

**FIGURE 1:**
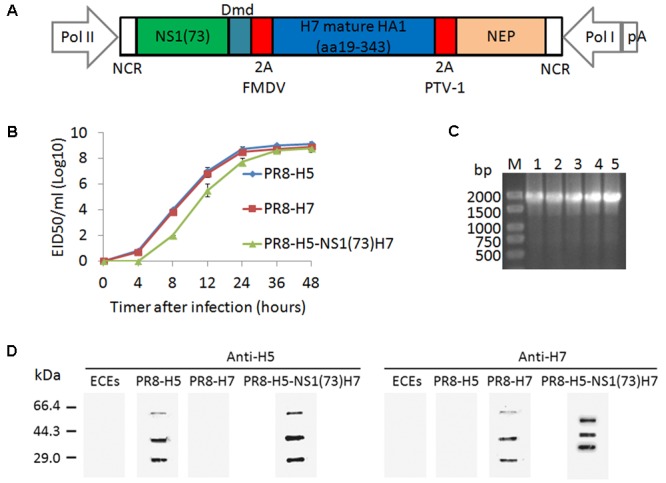
*In vitro* characterization of the PR8-H5-NS1(73)H7 virus. **(A)** Schematic representation of the promoters and coding sequences of the pHW-NS1(73)-H7 HA1-NEP plasmid used to generate the recombinant influenza virus. **(B)** The growth kinetics of PR8-H5-NS1(73)H7 and PR8-H5 were examined in ECEs. The allantoic fluids were collected and titrated in ECEs of each virus inoculated at 4, 8, 12, 24, 36, and 48 h post-infection with 10^4^ EID_50_. The data are presented as the mean and standard error of each data point. **(C)** Genetic stability of PR8-H5-NS1(73)H7 in chicken embryos, as determined with RT-PCR. (1) pHW plasmid encoding the NS1(73)-H7 HA1-NEP genes; (2) Passage 1 of PR8-H5-NS1(73)H7; (3) Passage 3 of PR8-H5-NS1(73)H7; (4); Passage 5 of PR8-H5-NS1(73)H7; (5) Passage 10 of PR8-H5-NS1(73)H7. **(D)** Uninfected ECEs and ECEs infected with PR8-H5 virus, PR8-H7 virus, or PR8-H5-NS1(73)H7 virus and the allantoic fluids were purified by ultracentrifugation through a 20% sucrose cushion. The proteins were visualized with western blotting and immune-detection with an anti-H5 or anti-H7 antibody.

In this study, a tri-cistronic NS-derived gene segment was designed in a single open reading frame consisting of NS1(1–73)Dmd, H7 HA1, and NEP separated from each other via two different 2A self-cleaving peptides. Two different 2A peptides were used to reduce the risk of recombination at these sites, as this could lead to the excision of the HA1 coding information. The 2A self-cleaving peptide of the FMDV was inserted between NS1(1–73)Dmd and HA1, while the latter was separated from NEP by the 2A self-cleaving peptide of PTV-1 (**Figure 1A**). The FMDV 2A peptide was the first 2A cleavage site to be described and has been since used for many applications, including the generation of recombinant influenza viruses (Ryan et al., 1991; Percy et al., 1994; Ryan and Drew, 1994; Basler et al., 2001). In addition, the PTV-1 2A peptide has been shown to have a high cleavage efficiency (Donnelly et al., 2001; Kim et al., 2011). This generated NS segment was used to rescue an influenza virus expressing dimeric NS1(1–73), HA1, and NEP in a PR8 virus genetic background (Hoffmann et al., 2002). On the basis of the HA and NA gene sequences of FJ/5 replacing the corresponding gene sequences of the PR8 virus, this rescue was successful and the resulting virus was named PR8-H5-NS1(73)H7.

### *In Vitro* Characterization of the PR8-H5-NS1(73)H7 Virus

To assess whether truncation of the NS1 gene or insertion of the HA1 gene affected viral fitness *in vitro*, the growth kinetics in ECEs were compared with the corresponding PR8 virus. First, a multi-cycle replication assay in ECEs was used to compare the growth kinetics of PR8-H5-NS1(73)H7 and the corresponding parental PR8-H5 and PR8-H7 viruses. During the initial passages in ECEs, PR8-H5-NS1(73)H7 had low infection and hemagglutination titers. However, the titers increased with the number of passages (**Table 1**). By the fifth passage, the infectious titer of PR8-H5-NS1(73)H7 was 8.76 ± 0.12 EID_50_/ml (log10). ECEs were inoculated with 10^4^ EID_50_ of each virus of the fifth passage and the virus titer in the allantoic fluid was determined at different time points after inoculation. On 4, 8, 12 h after infection, the endpoint titer of the PR8-H5-NS1(73)H7 virus was significantly lower than that of the parental PR8-H5 and PR8-H7 viruses (**Figure 1B**; *p* < 0.05), although their slope of the growth curve and the endpoint titer after 2 days were comparable (**Figure 1B**; *p* > 0.05), suggesting that the two recombinant viruses have similar growth kinetics.

**Table 1:** Infection and hemagglutination titers for the PR8-H5-NS1(73)H7 during passage in embryonated chicken eggs.

Passage	Mean EID_50_/ml (Log10) ± standard errors	Hemagglutination titer
1	6.36 ± 0.22	1:128
3	7.75 ± 0.14	1:256
5	8.76 ± 0.12	1:512

Examination of the NS1 gene with RT-PCR confirmed that PR8-H5-NS1(73)H7 retained the HA1 inserts in ECEs for 10 passages (**Figure 1C**). The size of the NS1 gene of the viral constructs containing the H7 HA1 protein corresponded to that amplified from the corresponding pHW plasmids (1770 bp). These results were confirmed by sequencing, which showed that the nucleotide sequences of the NS1 gene of PR8-H5-NS1(73)H7 corresponded to the H7 HA1 protein (data not shown).

To confirm HA1 expression, ECEs were inoculated with 10^4^ EID_50_ of the PR8-H5, PR8-H7, or PR8-H5-NS1(73)H7 virus and western blot analysis was performed. Detection of the PR8-H5 or PR8-H5-NS1(73)H7 virus with anti-H5 antibody revealed three major bands of approximately 63, 36, and 27 kDa (**Figure 1D**), which corresponded to the HA0, HA1, and HA2 proteins, respectively. Detection of the PR8-H7 virus with anti-H7 antibody revealed three major bands of approximately 63, 36, and 27 kDa (**Figure 1D**), which corresponded to the HA0, HA1, and HA2 proteins, respectively. Detection of the PR8-H5-NS1(73)H7 virus with anti-H7 antibody revealed three major bands of approximately 59, 46, and 36 kDa (**Figure 1D**). The 59-kDa band most likely corresponded to the uncleaved polyprotein (predicted size of 67.4 kDa) and the 36-kDa band to HA1. The middle 46-kDa band likely corresponded to the NS1Dmd-HA1 or the HA1-NEP fusion protein (predicted sizes of 52.9 and 52.2 kDa, respectively) (**Figure 1D**). The western blot results indicated that the cleavage at the 2A cleavage sites was incomplete. Taken together, these findings suggest that the PR8-H5-NS1(73)H7 virus replicates as efficiently as the parental PR8-H5 and PR8-H7 viruses *in vitro*, and infection of ECEs with the PR8-H5-NS1(73)H7 virus resulted in H7 HA1 expression, even though processing at the introduced 2A sites was incomplete.

### Protective Efficacy of the PR8-H5-NS1(73)H7 Vaccine

The PR8-H5-NS1(73)H7 vaccine was highly immunogenic in chickens. Three weeks after a single vaccination, the mean hemagglutinin inhibition (HI) antibody titers to the homologous H5 virus reached 8.5 ± 0.5 log2 (**Table 2**). The HI antibody titers induced by the PR8-H5-NS1(73)H7 vaccine to the H5 virus were comparable to that of the combination vaccine of PR8-H5 and PR8-H7.

**Table 2:** Efficacy of vaccines against clade 2.3.4.4 H5 and H7N9 highly pathogenic avian influenza viruses in chickens.

Vaccine group	Challenge virus	Mean HI titer 21 days after immunization (log2)	Virus shedding				Survival
			Day 3 p.c.^a^		Day 5 p.c.		
			Oropharyngeal	Cloacal	Oropharyngeal	Cloacal	
PR8-H5-NS1(73)H7	FJ/5	8.5 ± 0.5	0/10	0/10	0/10	0/10	10/10
	GD/RZ	5.2 ± 0.4	0/10	0/10	0/10	0/10	10/10
PR8-H5 + PR8-H7	FJ/5	8.7 ± 0.8	0/10	0/10	0/10	0/10	10/10
	GD/RZ	8.3 ± 0.5	0/10	0/10	0/10	0/10	10/10
PR8-H5	GD/RZ	<1.0	4/4 (4.9 ± 0.3)	4/4 (4.7 ± 0.5)	NA^b^	NA	0/10
PR8-H7	FJ/5	<1.0	4/4 (4.4 ± 0.5)	4/4 (4.5 ± 0.6)	NA	NA	0/10
Control	FJ/5	<1.0	4/4 (4.7 ± 0.5)	4/4 (4.9 ± 0.6)	NA	NA	0/10
	GD/RZ	<1.0	4/4 (4.5 ± 0.4)	4/4 (4.3 ± 0.4)	NA	NA	0/10
	ND^c^	<1.0	ND	ND	ND	ND	10/10

*^*a*^p.c., post challenge. ^*b*^NA, not applicable due to death of chickens. ^*c*^ND, not done.*

The mean HI antibody titers induced by the PR8-H5-NS1(73)H7 vaccine to the homologous H7 virus reached 5.2 ± 0.4 log2 (**Table 2**). Notably, the HI antibody titers induced by the PR8-H5-NS1(73)H7 vaccine to the H7 virus were significantly lower than that by the combination vaccine of PR8-H5 and PR8-H7 (*p* < 0.01).

Oropharyngeal and cloacal swabs were collected from each chicken on days 3 and 5 p.c. to assess viral shedding. All of the chickens vaccinated with the bivalent vaccine and the combination vaccine remained healthy challenge with the homologous HPAIV clade 2.3.4.4 H5 or H7N9 (**Table 2**). Viral shedding was not detected in any bird on days 3 or 5 p.c. However, univalent vaccine groups vaccinated with PR8-H5 (challenged with H7N9) and PR8-H7 (challenged with H5N6) and all of the control birds shed viruses and died within 4 days p.c. with HPAIV clades 2.3.4.4 H5 and H7N9 (**Table 2**). Moreover, the SPF chickens of the two groups immunized with the PR8-H5-NS1(73)H7 and PR8-H5 and PR8-H7 were of no significant difference (*p* > 0.05) in weight, as compared with the control chickens without challenge, during the experiments (data not shown).

## Discussion

To control the infection of H5 and H7N9 HPAIVs in China, since August 2017, the combination vaccine of H5 Re-8 and H7 Re1 has been used throughout China. A combination vaccine was prepared from a pool of allantoic suspensions of the H5 Re8 and H7 Re1 viruses, and then emulsified. Vaccine production requires more ECEs, as compared to a bivalent vaccine expressing both corresponding antigens.

In this study, replication competent recombinant influenza A virus of the subtype Í5N1 expressing the H7 HA1 protein was first constructed, which demonstrated that this vaccine could be used as a new candidate against AIV subtypes H5 and H7. Current killed influenza virus vaccines predominantly induce the production of anti-HA antibodies that specifically target antigenic sites in the globular head domain of the HA1 region and block receptor binding (Chiu et al., 2009; Hashem, 2015). Therefore, mature HA1 was chosen as the antigenic determinant and inserted into the NS segment between the NS1 and NEP sites. The construction strategy resembles that of De Baets et al. (2015) who inserted the green fluorescent protein (GFP) reporter into the NS1 segment of the PR8 virus. This strategy was adapted in an attempt to generate a virus expressing both the HA and NA proteins of H5N6 and the HA1 protein of H7N9. First, to avoid expression of an NS1-fusion protein, a tri-cistronic NS segment was designed that contained NS1(1–73)Dmd, H7 HA1, and NEP separated via two different 2A self-cleaving peptides. Although this strategy should theoretically produce three individual proteins, western blot of infected ECEs showed that cleavage was incompletely. Therefore, it would be interesting to also evaluate the cleavage efficiency of the two 2A self-cleaving peptides. Polyprotein processing could be improved by changing or optimizing one or both of the 2A auto-proteolytic cleavage peptides, as not all 2A self-cleaving peptides show the same cleavage efficiency (Percy et al., 1994).

Viruses expressing foreign genes ideally have high genetic stability, which is difficult to accomplish with engineered influenza viruses. The PR8-H5-NS1(73)H7 virus has similar replication kinetics *in vitro* as the parental PR8-H5 virus. Genome sequencing analysis showed that there was no mutation in the third, fifth, and tenth passage of the PR8-H5-NS1(73)H7 virus stock, indicating the stability of the PR8-H5-NS1(73)H7 virus. A previous study demonstrated that the NS1 gene sequence could tolerate insertions of foreign sequences exceeding its own length (Kittel et al., 2004). As far as we know, the 702-bp GFP gene was the longest fragment to be inserted to date. Here, we successfully inserted a 975-bp sequence of the mature H7 HA1 protein and obtained a stable virus.

Upon vaccination of SPF chickens with the inactivated PR8-H5-NS1(73)H7 vaccine, specific HI antibody responses were induced. At 21 days post-vaccination, the HI antibody titers against H5 induced by the PR8-H5-NS1(73)H7 vaccine were comparable to that by the combination vaccine of PR8-H5 and PR8-H7. However, the HI antibody titers against H7 by the PR8-H5-NS1(73)H7 vaccine were significantly lower than with the combination vaccine of PR8-H5 and PR8-H7 (*p* < 0.01). One reason may be that the PR8-H5-NS1(73)H7 virus expresses only the HA1 and no the HA2 stalk domain which could affect the conformation of HA protein (Stevens et al., 2006). The other reason may be the expression quantity of HA1 protein of H7 is little, although the HA1 is highly immunogenic (Du et al., 2011, 2013; Hessel et al., 2011; Lin et al., 2011). Nonetheless, the inactivated PR8-H5-NS1(73)H7 vaccine provided complete protection against challenge with both HPAIV H5N6 and H7N9.

In this study, we have successfully generated a construct expressing the HA1 subunit of H7 HA. It is expected that when making an inactivated virus vaccine only virions are typically in the preparations. In this case the virions will have displayed H5 HA on the surface. As the H7 HA1 has no transmembrane domain, it is completely unclear whether the H7 HA1 could be incorporated into the virion and how it would be delivered and how animals would induce an immune response against the inactivated virus vaccine. This does not change the observation that the immunization and challenge study demonstrated that the H7 HA1 was expressed and would be delivered to animals successfully as an inactivated vaccine. The mechanism needs further study.

## Conclusion

We generated a bivalent vaccine candidate PR8-H5-NS1(73)H7 expressing H5N6 HA and H7N9 HA1 of two AIV subtypes. This vaccine candidate induced obvious HI antibody titers against H5 and H7 antigens, although the antibody titers against H7 were significantly lower than that by the combination vaccine of PR8-H5 and PR8-H7 (*p* < 0.01). The bivalent vaccine PR8-H5-NS1(73)H7 provided complete protection against challenge with both HPAIV H5N6 and H7N9. These results indicate that the bivalent vaccine PR8-H5-NS1(73)H7 is highly immunogenic in chickens.

## Author Contributions

JL, GH, YW, and WJ conceived and designed the experiments and analyzed the data. JL, GH, SW, CP, XY, and WJ performed the experiments. WJ contributed reagents, materials, analysis tools and wrote the paper.

## Conflict of Interest Statement

The authors declare that the research was conducted in the absence of any commercial or financial relationships that could be construed as a potential conflict of interest.
